# The effect of rosmarinic acid on deformities occurring in brain tissue by craniectomy method. Histopathological evaluation of IBA-1 and GFAP expressions ^1^

**DOI:** 10.1590/s0102-865020200040000006

**Published:** 2020-06-19

**Authors:** Hüseyin Özevren, Engin Deveci, Mehmet Cudi Tuncer

**Affiliations:** I Associate Professor, Department of Neurosurgery , Faculty of Medicine , Dicle University , Diyarbakır , Turkey . Technical procedures, manuscript preparation and writing, final approval.; II PhD, Professor, Department of Histology and Embryology , Faculty of Medicine , Dicle University , Diyarbakır , Turkey . Technical procedures, histopathological examinations, manuscript preparation and writing, final approval.; III PhD, Professor, Department of Anatomy , Faculty of Medicine , Dicle University , Diyarbakır , Turkey . Technical procedures, histopathological examinations, manuscript preparation and writing, final approval.

**Keywords:** Rosmarinic Acid, Glial Fibrillary Acidic Protein, Brain Injuries, Traumatic, Rats

## Abstract

**Purpose:**

To investigate the role of Rosmarinic acid (RA) in the prevention of traumatic brain injury and the immunohistochemical analysis of IBA-1 and GFAP expressions.

**Methods:**

Healthy male rats were randomly divided into 3 groups consisting of 10 rats. Groups were as follows; control group, traumatic brain injury (TBI) group, and TBI+RA group. After traumatic brain injury, blood samples were taken from the animals and analyzed with various biochemical markers. And then IBA-1 and GFAP expressions were evaluated immunohistochemically.

**Results:**

Significant results were obtained in all biochemical parameters between groups. Immunohistochemical sections showed IBA-1 not only in microglia and macrophage activity but also in degenerative neurons in blood vessel endothelial cells. However, GFAP reaction and post-traumatic rosmarinic acid administration showed positive expression in astrocytes with regular structure around the blood vessel.

**Conclusion:**

Rosmarinic acid in blood vessel endothelial cells showed that preserving the integrity of astrocytic structure in the blood brain barrier may be an important antioxidant.

## Introduction

Traumatic brain injury is a health problem known as the cause of mortality and disability in young people. Primary and secondary injury cascades that cause delayed neuronal dysfunction, synapse loss and cell death are associated with traumatic brain injury ^1 , 2^ . Secondary damage develops within minutes to days following the primary insult, release of the inflammatory mediators, formation of the free radicals, excessive release of the neurotransmitters (glutamate and aspartate), influx of calcium and sodium ions into neurons, and dysfunction of mitochondria ^3^ .

Experimental models may lead to understanding the pathophysiology of repeated mild traumatic brain injury and identify potential therapeutic targets. Adult models of repeated mild closed head impact and blast injury have shown long-term neurocognitive dysfunction associated with traumatic axonal injury and microglial activation ^4 - 6^ .

Rosmarinic acid is a naturally occurring polyphenolic antioxidant found in numerous common herbal plants. RA is isolated from herbal balm mint plants, including Melissa officinalis, Rosmarinus officinalis, and Prunella vulgaris ^7 - 9^ . Rosmarinic acid has demonstrated to have antioxidant, anticarcinogenic, anti-inflammatory, antidepressant, and antimicrobial effects ^10 - 13^ . Rosmarinic acid has shown to be neuroprotective and induce neuroprotection from reactive glial cells in in vitro Alzeimer patient models, spinal cord injury and Parkinson’s disease by inhibiting nitric oxide (NO) production ^14 - 16^ .

Glial fibrillary acidic protein (GFAP) is an intermediate filament protein found in the skeleton of astroglia. Data from studies have shown that increased local tissue GFAP immunoreactivity is a sensitive indicator of neuronal damage and its increase is considered to be a determinant of reactive astrocytosis. GFAP level in blood fluid increases when cerebral tissue or spinal cord cells are damaged due to trauma or disease ^17^ . The molecule with ionized calcium binding adapter protein-1 (IBA-1) is a structurally expressed 17-kDa actin binding protein specific for all microglia. Anti Iba-1 is particularly reactive to microglia and macrophages, and brain tissues and cell culture are characterized in combination with GFAP monoclonal antibody reacting to specific astrocytes.

Traumatic brain injury is a damage associated with disruption of the blood-brain barrier, and the role of rosmarinic acid in preventing this damage and the immunohistochemical examination of IBA-1 and GFAP expressions were aimed.

## Methods

All techniques performed in this examination were approved by the Ethics Committee for Animal Experimentation of the Faculty of Medicine at Dicle University, Turkey. Male Sprague Dawley rats (280–310 g) were housed in an air-conditioned room with 12-h light and dark cycles, where the temperature (23±2°C) and relative humidity (65–70%) were kept constant. All rats at the end of experiment were healthy and no difference in food/water consumption and body weight gain between experimental and control rats were observed.

### Traumatic brain injury model and sedation procedure

The animals were anesthetized by an intraperitoneal injection of 5 mg/kg xylazine HCl (Rompun, Bayer Health Care AG, Germany) and 40 mg/kg ketamine HCl (Ketalar, Pfizer Inc., USA), and were allowed to breathe spontaneously. A rectal probe was inserted, and the animals were positioned on a heating pad that maintained the body temperature at 37°C.

Three groups (10 rats per group) were arranged as below:

1. **Control group:** Isotonic saline solution was administered i.p. for 7 days.
2. **TBI group:** The most commonly used and accepted traumatic brain injury methods were also modified in this study ^18 , 19^ .

### Calvarial defect procedure

The trauma device consisted of a unique column of a plexiglass tube with a freely falling steel weight by gravity onto a metallic helmet fixed by skull vertex of the rat by bonewax. The steel weight falls through a 1-m vertical section of the plexiglass tube held in place with ring a stand. The internal surface of the tube was plastered with a thin lubricant. A 2-mm thick steel disc was used as the helmet. The scalp of each of the anesthetized rat was shaved, a midline incision was performed and the periosteum was retracted. The metallic disc was fixed to the central portion of skull by using bonewax. The animals were placed in a prone position on a foam bed. The lower end of the plexiglas tube was then positioned directly above the helmet. The injury was delivered by dropping the designated weight from a predetermined height. An inflexible rope was tied to the weight to prevent repeated impacts.

1. **TBI+RA group:** Rosmarinic acid administered in the dose of 50 mg/kg via oral gavage for 7 days after the trauma.

After seven days, all animals were sacrificed by an intraperitoneal injection of 5 mg/kg xylazine HCl (Rompun, Bayer HealthCare AG, Germany) and 40 mg/kg ketamine HCl (Ketalar, Pfizer Inc, USA). After traumatic brain injury, blood samples were taken from the animals and analyzed with various biochemical markers for MDA, GSH-Px, MPO and evans blue assay for blood–brain barrier permeability values. Then, left parietal lobes of the brain cortex were rapidly removed. For the histological examination, brain tissues were fixed in 10% formaldehyde solution, post-fixed in 70% alcohol, and embedded in paraffin wax. The sections were stained with Hematoxylin-Eosin. Hematoxylin-Eosin staining method is detailed below.

### Hematoxylin-eosin staining procedure

1. Deparaffinize sections, 2 changes of xylene, 10 minutes each.
2. Re-hydrate in 2 changes of absolute alcohol, 5 minutes each.
3. 95% alcohol for 2 minutes and 70% alcohol for 2 minutes.
4. Wash briefly in distilled water.
5. Stain in Harris hematoxylin solution for 8 minutes.
6. Wash in running tap water for 5 minutes.
7. Differentiate in 1% acid alcohol for 30 seconds.
8. Wash running tap water for 1 minute.
9. Bluing in 0.2% ammonia water or saturated lithium carbonate solution for 30 seconds to 1 minute.
10. Wash in running tap water for 5 minutes.
11. Rinse in 95% alcohol, 10 dips.
12. Counterstain in eosin-phloxine solution for 30 seconds to 1 minute.
13. Dehydrate through 95% alcohol, 2 changes of absolute alcohol, 5 minutes each.
14. Clear in 2 changes of xylene, 5 minutes each.
15. Mount with xylene based mounting medium.

### Malondialdehyde and glutathione peroxidase assays

Malondialdehyde (MDA) levels and glutathione peroxidase (GSH-Px) activities were determined in the left parietal lobe of each rat, and the average values of each group were calculated. Each sample was prepared as a 10% homogenate (according to weight) in 0.9% saline using a homogenizer on ice. Then, the homogenate was centrifuged at 2000 rpm for 10 min, and the supernatant was collected. MDA levels were determined using the double heating method of Draper and Hadley ^20^ . MDA is an end product of fatty acid peroxidation that reacts with thiobarbituric acid (TBA) to form a coloured complex. Briefly, 2.5 mL of TBA solution (100 g/L) was added to 0.5 mL of homogenate in a centrifuge tube, and the tubes were placed in boiling water for 15 min. After cooling with flowing water, the tubes were centrifuged at 1000 rpm for 10 min, and 2 mL of the supernatant was added to 1 mL of TBA solution (6.7 g/L); these tubes were placed in boiling water for another 15 min. After cooling, the amount of TBA-reactive species was measured at 532 nm, and the MDA concentration was calculated using the absorbance coefficient of the MDA–TBA complex. MDA values were expressed as nanomoles per gram (nmol/g) of wet tissue. The GSH-Px activity was measured by the method of Paglia and Valentine ^21^ . An enzymatic reaction was initiated by the addition of hydrogen peroxide (H2O2) to a tube that contained reduced nicotinamide adenine dinucleotide phosphate, reduced glutathione, sodium azide, and glutathione reductase. The change in absorbance at 340 nm was monitored by spectrophotometry. Data were expressed as U/g protein.

### Tissue myeloperoxidase activity

Myeloperoxidase (MPO) activity in tissues was measured by a procedure similar to that described by Hillegass *et al* . ^22^ . MPO is expressed as U/g tissue.

### Evans blue assay for blood–brain barrier permeability

To evaluate the blood-brain barrier integrity, Evans blue dye was used as a marker of albumin extravasation. To evaluate the blood–brain barrier permeability, Evans blue (EB) dye was used as a marker of albumin extravasation. Briefly, EB dye (2% in saline, 4 ml/kg) was injected via the jugular vein 48 hours after the induction of trauma and it was allowed to remain in circulation for 30 minutes. At the end of experiments, the chest was opened and rats were perfused transcardiacally with 250 ml of saline at a pressure of 110 mmHg for 15 minutes until the fluid from the right atrium became colorless. After decapitation, the brain was removed. Then, cortex was weighed for quantitative measurement of EB-albumin extravasation. Brain samples were homogenized in 2.5 ml phosphatebuffered saline (PBS) and mixed by vortexing for 2 minutes after the addition of 2. 5 ml of 60% trichloroacetic acid, to precipitate the protein. Samples were cooled and then centrifuged for 30 minutes at 1000 x g. The supernatant was measured at 620 nm for absorbance of EB using a spectrophotometer. Evans blue was expressed as μg/mg of brain tissue against a standard curve ^23^ .

### Immunohistochemical technique

Formaldehyde-fixed tissue was embedded in paraffin wax for further immunohistochemical examination. Sections were deparaffinized in %96 alcohol. The antigen retrieval process was performed twice in citrate buffer solution (pH 6.0), first for 10 min, and second for 5 min, boiled in a microwave oven at 700 W. They were allowed to cool to room temperature for 20 min and washed twice in distilled water for 5 min. Endogenous peroxidase activity was blocked in 0.1% hydrogen peroxide (catalogue #TA-015-HP, Thermo Fisher Scientific, US) for 25 min. Ultra V block (TA-125-UB, Thermo Fisher Scientific, US) was applied for 10 min prior to the application of primary antibodies, which were left on overnight IBA-1 antibody (1:100 dilution) (Catalog # PA5-18039, Thermo Fisher Scientific, US) and Glial fibrillary acidic protein (GFAP) antibody (1:100 dilution) (Catalog # PA1-10019, Thermo Fisher Scientific, US). The sections were washed 3 times for 5 min in PBS and then were incubated with biotinylated secondary antibody (catalogue #TP-125-BN, Thermo Fisher Scientific, US) for 20 min. After washing with PBS, streptavidin peroxidase (catalogue #TS-125-HR, Thermo Fisher Scientific, US) was applied to the sections for 20 min. The sections were washed 3 times for 5 min in PBS. Diaminobenzidine (catalogue #TA-012-HDC, Thesermo Fisher Scientific, US) was applied to the sections for up to 20 min as a chromogen. The control slides were prepared using the same procedure, without primary antibodies. Counterstaining was done using Harris’s haematoxylin for 45 s, dehydrated through ascending alcohol and cleared in xylene (Product Number: HHS32 Sigma, hematoxylin solution, Harris modified, Sigma-Aldrich, 3050 Spruce Street, Saint Louis, MO, 63103, USA). The slides were mounted with Entellan ^®^ (lot: 107961, Sigma-Aldrich, St. Louis, MO, USA) and examined under a light microscope (Olympus, Germany).

### Statistical analysis

Statistical analysis was carried out using *GraphPad Prism 4.0 software* (GraphPad Software, 2003, San Diego, CA, USA). All data are presented as mean ± standard deviation. The data of the parameters were evaluated with the non-parametric Kruskal–Wallis test, and then Bonferroni Correction was performed with the comparative Mann-Whitney U test between the groups (Tables 1 and 2). Histological scores were assessed by two independent, blinded investigators who observed two sections per male rat at magnifications of x10 and x100.

## Results

### Biochemical findings

MDA values in the trauma (TBI) group were significantly higher than those of the control group (p<0.001) while the TBI+ Rosmarinic acid group had significantly lower levels than those of the trauma (TBI) group (p<0.001). When the tissue MPO activities of the control group were compared with that of the TBI group, a statistically significant difference was observed (p < 0.01); these data showed that after TBI, tissue MPO activity was increased. A significant decrease was observed in TBI group after TBI when compared with control. (p < 0.001) ( Table 1 ).

**Table 1:** Biochemical results relevant to the study groups.

	Control	TBI	TBI+RA
MDA (nmol/g)	*33.12±2.74*	40.32±3.65***	*36.29±2.79* ^++^ *
GSH (µmol/g)	*1.41±0.31*	0.8±0.18***	*1.09±0.26* ^++^ **
MPO (U/g)	*3.7±0.87*	5.82±0.91***	*4.73±0.76* ^++^ **
Edema (%)	*60.78±5.02*	67.73±6.07*	*65.49±7.32*

Values are represented as mean ± SD. Each group consists of ten rats.

*** p<0.001, *vs.* control

** p<0.01, *vs.* control

* p<0.05, *vs.* control

++p<0.01, *vs.* trauma

+p<0.05, *vs.* trauma

Data are expressed as the mean ± standard deviation and mean rank. The quantification of all parameters: **0:** no change, **1:** too week, **2:** week, **3:** middle, **4:** strong. (While scoring was carried out, 10 different areas were scanned for each section and the average of 15 cells selected randomly was obtained and the average score of the related preparation was obtained. Decimal digits are converted to integers when obtaining averages before statistical analysis). The data of the parameters were evaluated with the non-parametric Kruskal–Wallis test, and then Bonferroni Correction was performed with the comparative Mann-Whitney U test between the groups. A significant decrease in the values between the groups was significant and significant (*P=0 with Kruskal-Wallis test , **P < 0.05 with Mann–Whitney U-test with a Bonferroni correction) ( Table 2 ).

**Table 2:** Histopathological scoring and Immunohistochemistry expression values.

Parameter	Groups	n	Mean±SD	Mean Rank	Kruskal-Wallis Test value	Multiple comparisons for groups (p<0.05)
Dilatation in blood vessels	*(1) Control group*	*10*	0.40±0.51	*6.70*	23.839 *P=0	**(2)
	*(2) TBI group*	*10*	3.30±0.67	*25.30*		**(1) **(3)
	*(3) TBI+RA group*	*10*	1.40±0.51	*14.50*		**(2)
Inflamation	*(1) Control group*	*10*	0.40±0.51	*6.30*	22.640 *P=0	**(2) **(3)
	*(2) TBI group*	*10*	3.20±0.78	*24.60*		**(1)
	*(3) TBI+RA group*	*10*	1.70±0.67	*15.60*		**(1)
Degeneration in endothelial cells	*(1) Control group*	*10*	0.60±0.51	*5.50*	21.660 *P=0	**(2) **(3)
	*(2) TBI group*	*10*	2.40±0.51	*21.00*		**(1)
	*(3) TBI+RA group*	*10*	2.30±0.48	*20.00*		**(1)
Apoptosis in microglia	*(1) Control group*	*10*	0.30±0.48	*5.80*	24.426 *P=0	**(2) **(3)
	*(2) TBI group*	*10*	3.10±0.56	*24.70*		**(1)
	*(3) TBI+RA group*	*10*	1.90±0.56	*16.00*		**(1)
İBA-1 expression	*(1) Control group*	*10*	1.80±0.63	*8.00*	16.899 *P=0	**(2) (3)**
	*(2) TBI group*	*10*	3.20±0.42	*22.70*		**(1)
	*(3) TBI RA group*	*10*	2.50±0.52	*15.80*		**(2)
GFAP expression	*(1) Control group*	*10*	2.80±0.63	*15.40*	8,427 *P=0,015	**(2) **(3)
	*(2) TBI group*	*10*	3.30±0.67	*20.80*		**(3)
	*(3 TBI+RA group*	*10*	2.30±0.42	*10.30*		**(2)

### Histopathologic examinations

#### In the histopathologic examinations findings were as follows for each group

1. **Control group:** Neural and vascular structures had normal anatomical structure in HE sections (Fig. 1a).
  **Figure:**
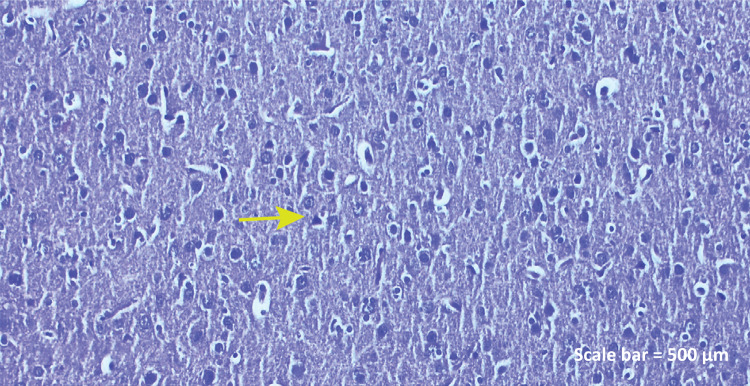
Figure 1a - Haematoxylin-eosin staining (Control group). Histopathologic examination revealed regular distribution of pyramidal ( *yellow arrow* ) and oval neurons, marked nuclei, solitary glial cells, and endothelial cells of blood vessels ( *left parietal lobe* ). Scale bar = 500 μm.
2. **TBI group:** Pyramidal neurons belonging to some cerebral cortex had picnosis in nuclei and apoptosis in glia cells. When vascular structures were evaluated, dilatation and obstruction in blood vessels, hyperplasia in endothelial cells, mononuclear cell leaks in small clusters were observed (Fig. 1b).
  **Figure:**
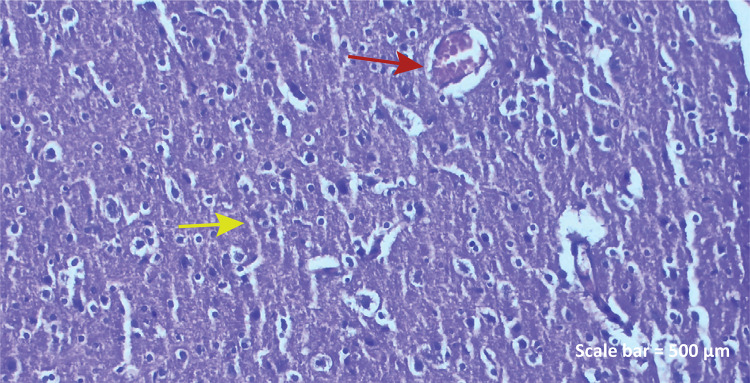
Figure 1b - Haematoxylin-eosin staining (TBI group) Some pyramidal neurons showed picnosis in nuclei, dilatation and congestion in blood vessels ( *red arrow* ), hyperplasia in endothelial cells, apoptotic changes in glia cell nuclei, and mononuclear cell infiltrations in small clusters around the vessel ( *yellow arrow* ) ( *left parietal lobe* ). Scale bar = 500 μm.
3. **TBI+RA group:** In the group treated with rosmaniric acid, while polygonal and polyhedral neurons appear hyperplasic, apoptotic changes in glia cells are reduced. There was also a decrease in bleeding and inflammatory cells (Fig. 1c).
  **Figure:**
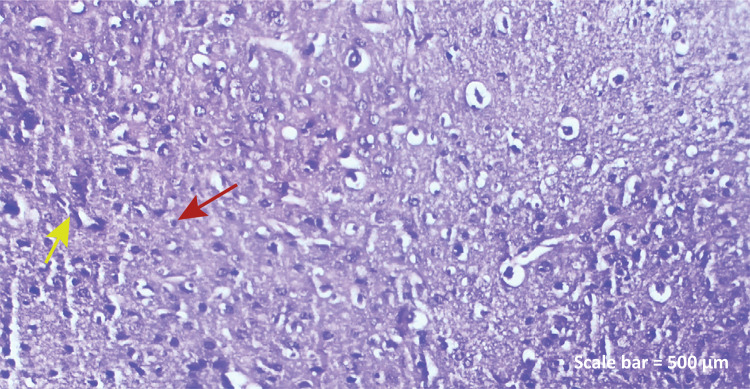
Figure 1c - Haematoxylin-eosin staining (TBI+RA group) Polygonal and polyhedral neurons appear hyperplasic ( *yellow arrow* ), apoptotic changes in glia cells decrease ( *red arrow* ), narrowing of lumen of blood vessels, decrease in bleeding and decrease in inflammatory cells ( *left parietal lobe* ). Scale bar = 500 μm.

## Immunohistochemical examinations

1. **Control group:** While IBA-1 positive expression was seen in microglia cells, blood vessel endothelial cells (Fig. 2a), positive GFAP expression was seen in astrocyte cell feet and some glia cell nuclei around small capillaries of the brain cortex (Fig. 3a).
  **Figure:**
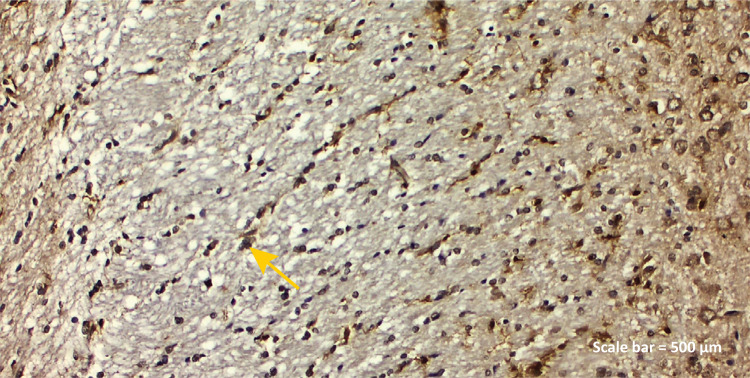
Figure 2a - IBA-1 immunostaining (Control group). IBA-1 positive expression was seen in microglia cells, blood vessel endothelial cells and nuclei of some neurons ( *yellow arrow* ) ( *left parietal lobe* ). Scale bar = 500 μm.
  **Figure:**
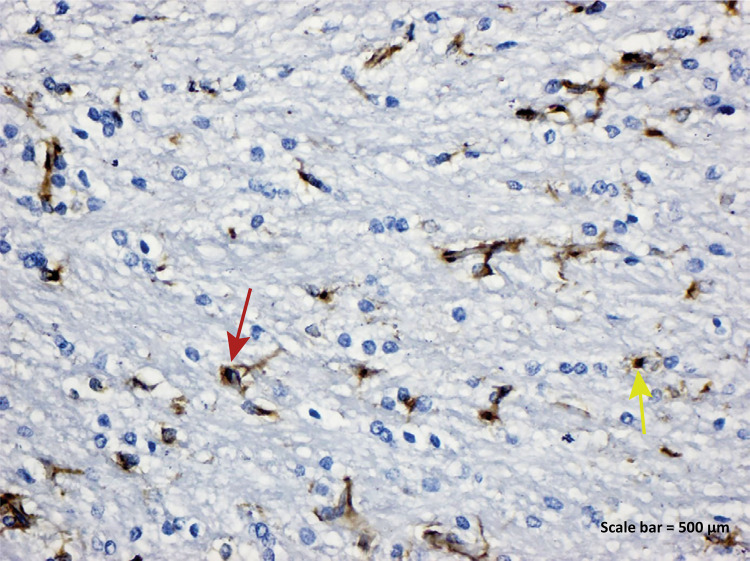
Figure 3a - GFAP immunostaining (Control group). Positive GFAP expression was seen in astrocytes cell feet ( *red arrow* ) and some glia cell nuclei around the small capillary vessels in the brain cortex ( *left parietal lobe* ). Scale bar = 500 μm.
2. **TBI group:** While positive IBA-1 expression was observed in microglia cells and degenerative neurons (Fig. 2b), GFAP reaction was detected in apoptotic glia cells (Fig. 3b).
  **Figure:**
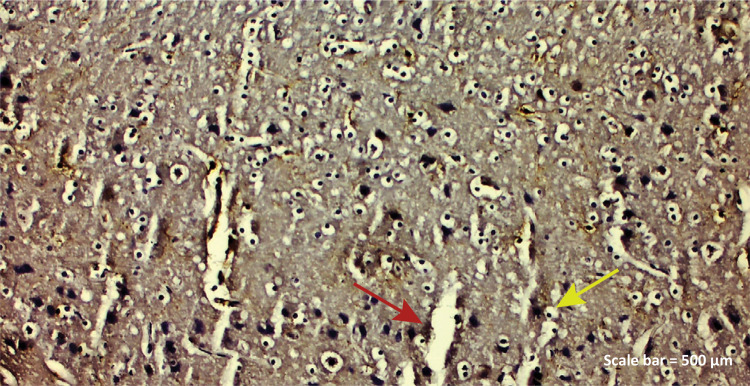
Figure 2b - IBA-1 immunostaining (TBI group). Positive IBA-1 expression was observed in hyperplasia endothelial cells ( *red arrow* ), microglia cells and degenerative neurons in the blood vessel ( *yellow arrow* ) lumen ( *left parietal lobe* ). Scale bar = 500 μm.
  **Figure:**
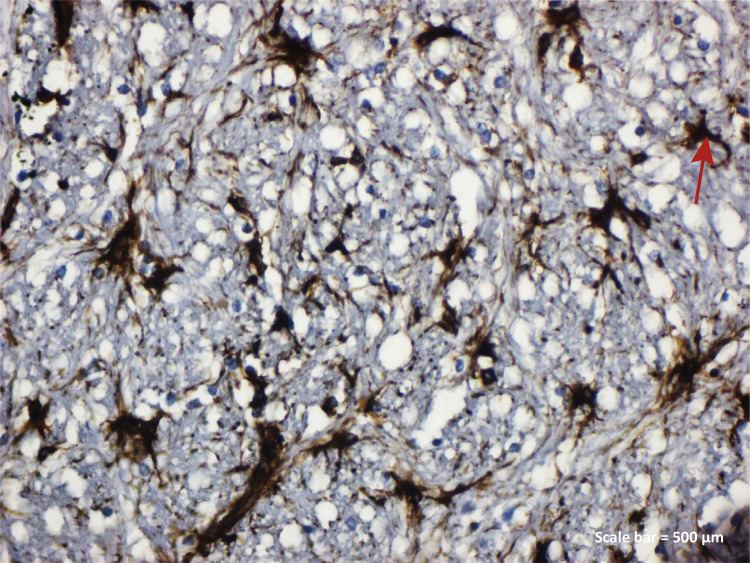
Figure 3b - GFAP immunostaining (TBI group). GFAP reaction of apoptotic glia cells, irregularities and shortened extensions of astrocytes ( *red arrow* ) were observed with blood vessel dilatation ( *left parietal lobe* ). Scale bar = 500 μm.
3. **TBI+RA group:** IBA-1 expression was found to be significant in endothelial cells and small microglia cells in blood vessels (Fig. 2c). In the other group, astrocytes in the blood vessels were regular and GFAP expression was positive in these cells (Fig. 3c).
  **Figure:**
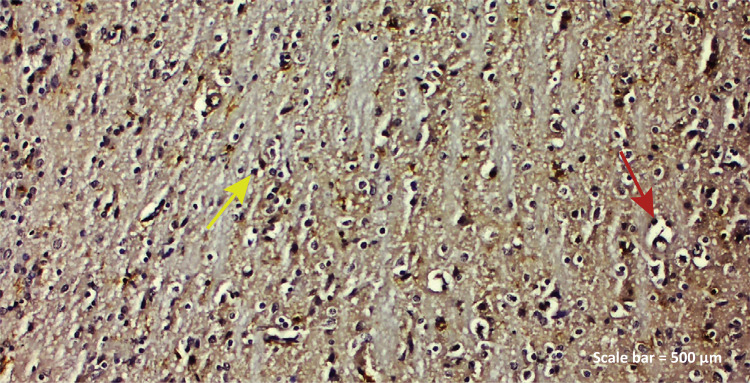
Figure 2c - IBA-1 immunostaining (TBI+RA group). IBA-1 expression was found to be significant in endothelial cells in blood vessels ( *red arrow* ) and small microglia cells ( *yellow arrow* ) ( *left parietal lobe* ). Scale bar = 500 μm.
  **Figure:**
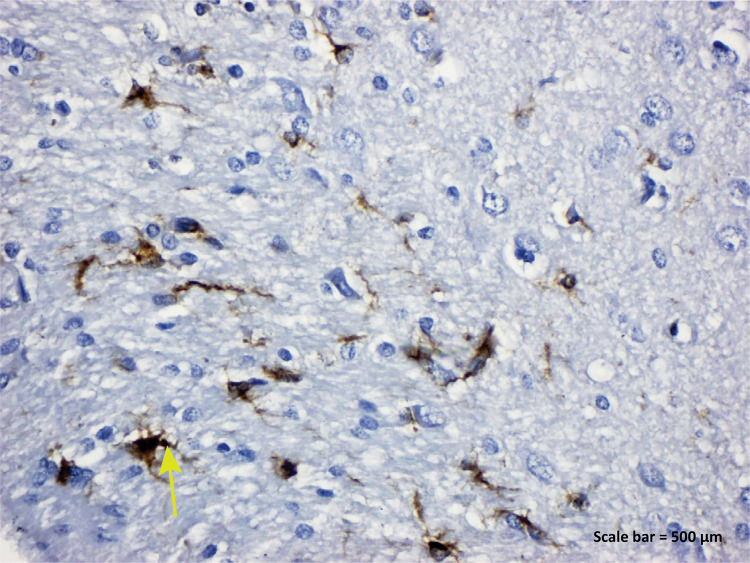
Figure 3c - GFAP immunostaining (TBI+ RA group). Regular astrocytes in blood vessels and GFAP expression was found to be positive ( *yellow arrow* ) ( *left parietal lobe* ). Scale bar = 500 μm.

## Discussion

Traumatic brain injury often promotes disruption of the blood-brain barrier integrity and the neurovascular unit, which can result in vascular leakage, edema, hemorrhage, and hypoxia. Other pathologic mechanisms include cell death within the meninges and brain parenchyma, stretching and tearing of axonal fibers, and disruptions at the junctions between white and gray matter, stemming from rotational forces that cause shearing injuries ^24^ . Previous studies have shown that a variety of pathological factors, such as oxidative stress, inflammatory response and apoptosis, are involved in secondary brain injury after traumatic brain injury. Furthermore, early interventions to reduce the level of oxidative stress and the extent of the inflammatory response can significantly reduce the extent of traumatic brain injury ^25^ . Özevren *et al* . ^26^ found enlarged blood vessels, bleeding and swelling after traumatic injury in the brain. In addition, nuclei of the neurons were dissociated and vacuolar degeneration was observed. Furthermore, early interventions to reduce the level of oxidative stress and the extent of the inflammatory response can significantly reduce the extent of TBI ^17^ .

MDA, a toxic product of lipid peroxidation, has been reported to promote cross-linking of nucleic acids, proteins and phospholipids, which cause dysfunction of macromolecules ^27^ , and MDA levels have been reported to function as a key marker of lipid peroxidation ^28^ . SOD, an endogenous antioxidant enzyme, converts harmful superoxide radicals into hydrogen peroxide, therefore, a superoxide radical scavenger to provide cytop protection against damage caused by toxic oxygen-free radicals. Oxidative stress, inflammatory response and various pathological factors such as apoptosis and changes in vascular structure have been involved in secondary brain injury after traumatic brain injury. It has been reported that this mechanism reduces the level of oxidative stress, early interventions and inflammatory response, and plays an important role in the degree of traumatic brain injury ^25^ . In our study, it was found statistically that brain edema was higher in the group that caused traumatic brain injury. However, it was seen that there was no significant effect in the group administrated rosmaniric acid ( Table 1 ).

Baloğlu *et al* . ^29^ reported a significant increase in MDA levels and a significant decrease in GSH and MPO levels in spinal cord injury. Çetin *et al* . ^30^ reported an increase in MDA levels in traumatic brain injury, GSH-Px levels of the control group were significantly higher than the trauma level, and MPO levels of the control group were significantly lower than the trauma levels. In our study, MDA.GSH, MPO values in the control and TBI group were similar to those of Cetin *et al* . ^30^ In the application of rosmarinic acid, it was thought that rosmarinic acid, which is an antioxidant, could have an effect on the regulation of oxidative stress determinants with the values close to MDA, GSH, MPO control group ( Table 1 ).

GFAP is closely related to the other three non-epithelial type III IF family members, vimentin, desmin and peripherin, which are all involved in the structure and function of the cell’s cytoskeleton. GFAP is thought to help to maintain astrocyte mechanical strength as well as the shape of cells, but its exact function remains poorly understood, despite the number of studies using it as a cell marker. Nevertheless, GFAP, a brainspecific protein that acts as the major integral component of the cell skeleton of astrocytes, after brain injury discharges the brain cells into the interstitial fluid in the environment and causes deterioration in the blood-brain barrier ^31^ . An increase in GFAP expression is a cardinal feature of many pathological conditions of the central nervous system and astrocytes. Increasing numbers of GFAP positive expression astroglia cells following TBI have been described in several experimental studies in animals. GFAP was positively expressed in the normal brain tissue; process in astrocytes around the blood brain barrier rupture was defined as significant GFAP expression ^30 , 32^ . In the TBI group of our study, it was observed that apoptotic glia cells were positive in GFAP reaction with a significant irregularity in astrocytes and shortened extensions in blood vessel dilatations, while GFAP protein, an important marker for astrocytes, increased due to changes in the blood brain barrier due to traumatic brain damage (Fig. 3b). GFAP reaction was positive in astrocytes with regular structure around blood vessels by post-traumatic rosmarinic acid administration (Fig. 3c).

Rosmarinic acid has many biological activities, including neuroprotective, antioxidant and anti-inflammatory effects. Rocha *et al* . ^33^ observed that administration of rosmarinic acid and extract at the dose of 25 mg/kg reduced paw oedema at 6 hr by over 60%, exhibiting a dose-response effect, suggesting that rosmarinic was the main contributor to the anti-inflammatory effect. The neuroprotective effects of rosmarinic acid against nitrosive stress in vivo have been reported to reduce Nuclear Factor kappa B and inhibit NO production from active glial cells. Rosmarinic acid is said to relieve nitrosative stress through two mechanisms. First, it inhibits the production of NO from macrophages and microglia. The other acts directly as reactive nitrogen species cleaners, including NO ^34^ . The important role played by activated astrocytes is probably related to cytokine production after injury ^35^ . It was also suggested that GFAP up-regulation after injury has a role in the maintenance of neuropathic pain ^36 , 37^ . An earlier investigation reported that up-regulation of GFAP was observed 3 days after nerve injury and lasted until day 21 ^38^ . In this study, it was thought that IBA-1 had a down-regulated expression, but the positive effect of rosmarinic acid and IBA-1 activity was important in balancing microglia activation.

Ohsawa *et al* . ^39^ have shown that the IBA-1 protein, which is related to the calcium binding signaling pathway, may be responsible for cell migration and phagocytic activity of the microglia - macrophage. It has been reported that upregulation of IBA-1 in the active microglia in the peri-ischemic area contributes to cell migration and that IBA-1 protein in brain macrophages may be involved in phagocytic activity. In another study, activation of IBA-1 positive microglia and brain macrophages has been demonstrated prominently in both the ischemic nucleus and the peri-ischemic area ^40^ . In the TBI group of our study, IBA-1 protein was found to be positive in endothelial cells, microglia cells and degenerative neurons in the blood vessel lumen (Fig. 2b). As we know well in among brain cells, the IBA-1 gene is strongly and specifically expressed in microglia. Circulating macrophages also express IBA-1. In the light of this information, we aimed to show that IBA-1 was found to be determinant not only in microglia and macrophage activity but also in blood vessel endothelial cells degenerative neurons with rosmarinic acid administration (Fig. 2c). According to our findings, it was thought that the increase of IBA-1 expression in endothelial cells and microglia cells in the group treated with rosmarinic acid was important in determining some reactions with antioxidative, anti-inflammatory and angiogenetic effects such as rosmarinic acid.

## Conclusions

Despite the results, there are a few limitations in the clinical contribution. Especially after trauma, GFAP increases in intermediate filaments in astrocytes with the development of astrocytosis in the infection or neurodegenerative process and induction in the signal pathway in the astrocytes. Due to the antioxidative effect of rosmarinic acid, it was thought that it may regulate this protein increase in astrocyte regulation. TBI caused cell apoptosis, microglial activation and an inflammatory response. The weakening of microglial activation, Iba-1, an important biomarker for microglial activation, is thought to have its expression down-regulated, but the positive Iba-1 activity with the effect of rosmarinic acid is important to balance microglia activation.
